# Translational Application of Microfluidics and Bioprinting for Stem Cell-Based Cartilage Repair

**DOI:** 10.1155/2018/6594841

**Published:** 2018-02-20

**Authors:** Silvia Lopa, Carlotta Mondadori, Valerio Luca Mainardi, Giuseppe Talò, Marco Costantini, Christian Candrian, Wojciech Święszkowski, Matteo Moretti

**Affiliations:** ^1^Cell and Tissue Engineering Laboratory, IRCCS Galeazzi Orthopaedic Institute, Milan, Italy; ^2^Department of Electronics, Information and Bioengineering, Politecnico di Milano, Milan, Italy; ^3^Regenerative Medicine Technologies Lab, Ente Ospedaliero Cantonale (EOC), Lugano, Switzerland; ^4^Laboratory of Biological Structures Mechanics-Chemistry, Material and Chemical Engineering Department “Giulio Natta”, Politecnico di Milano, Milan, Italy; ^5^Department of Chemistry, Sapienza University of Rome, Rome, Italy; ^6^Unità di Traumatologia e Ortopedia-ORL, Ente Ospedaliero Cantonale (EOC), Lugano, Switzerland; ^7^Faculty of Materials Science and Engineering, Warsaw University of Technology, Warsaw, Poland; ^8^Swiss Institute for Regenerative Medicine, Lugano, Switzerland

## Abstract

Cartilage defects can impair the most elementary daily activities and, if not properly treated, can lead to the complete loss of articular function. The limitations of standard treatments for cartilage repair have triggered the development of stem cell-based therapies. In this scenario, the development of efficient cell differentiation protocols and the design of proper biomaterial-based supports to deliver cells to the injury site need to be addressed through basic and applied research to fully exploit the potential of stem cells. Here, we discuss the use of microfluidics and bioprinting approaches for the translation of stem cell-based therapy for cartilage repair in clinics. In particular, we will focus on the optimization of hydrogel-based materials to mimic the articular cartilage triggered by their use as bioinks in 3D bioprinting applications, on the screening of biochemical and biophysical factors through microfluidic devices to enhance stem cell chondrogenesis, and on the use of microfluidic technology to generate implantable constructs with a complex geometry. Finally, we will describe some new bioprinting applications that pave the way to the clinical use of stem cell-based therapies, such as scaffold-free bioprinting and the development of a 3D handheld device for the in situ repair of cartilage defects.

## 1. Introduction

Cartilage defects, due to trauma or progressive joint degeneration, can impair the most elementary daily activities, such as walking or running. Due to the limited self-repair ability of cartilage, these lesions can easily evolve into osteoarthritis (OA), leading to the complete loss of articular function and to the subsequent need for joint replacement [1]. In the last decades, the limitations of standard surgical treatments for cartilage repair have triggered the development of cell-based therapies. Autologous chondrocyte implantation (ACI) has been the first cell-based approach to treat cartilage defects [2, 3], and more lately, stem cells have been proposed as an alternative cell source for cell-based cartilage repair [4, 5]. Among the various types of adult stem cells, mesenchymal stem cells derived from bone marrow (BMSCs) have been widely used for cartilage applications due to their well-demonstrated chondrogenic potential [6, 7]. Besides BMSCs, more lately, adipose-derived mesenchymal stem cells (ADMSCs) obtained from different adipose depots, including knee infrapatellar fat pad, have gained growing interest as an alternative cell source for cartilage repair [8–10].

In the development of stem cell-based therapies for tissue regeneration, bioprocessing optimization is required to exploit the remarkable potential of stem cells. In particular, efficient cell differentiation protocols and the design of proper biomaterial-based supports to deliver cells to the injury site need to be addressed and overcome through basic and applied research [11]. In this scenario, microfluidic systems have attracted significant interest implementing platforms, in which the control of local environmental conditions, including biochemical and biophysical parameters, is exploited to study and direct stem cell fate [12, 13]. Indeed, microfluidic technology enables the precise control over fluids at the microscale, thus allowing mimicking of the natural cell microenvironment by continuous perfusion culture or by creating chemical gradients [14]. Because of these features, microfluidic devices can be efficiently used to investigate the plethora of factors that guide stem cell differentiation towards a specific cell lineage, testing several conditions with minimal requirements in terms of cell number and amount of reagents to perform large experiments [15]. So far, a suite of microfluidic devices has been developed to investigate the influence of both biochemical and biophysical factors on stem cell differentiation in order to outline new protocols for stem cell chondrogenesis [16–18]. Recently, microfluidic technology has also been used to fabricate advanced systems for 3D bioprinting to produce microchanneled scaffolds for the enhancement of nutrient supply [19] or to encapsulate cells within microspheres or fibers [20–22]. 3D bioprinting is a novel research field that is showing excellent potential for the development of engineered tissues, allowing the fabrication of heterogeneous constructs with biochemical composition, mechanical properties, morphology, and structure comparable to those of native tissues [23, 24]. As reported in several recent reviews [23, 25–28], this technology has the potential to overcome major problems related to the clinical translation of tissue engineering products for cartilage repair, which has been so far limited due to the poor results obtained in terms of construct functionality. Indeed, cartilage properties are determined by its complex architecture characterized by anisotropic orientation of collagen fibers and density gradients of chondrocytes, which even express slightly different phenotypes [29, 30]. 3D bioprinting, due to its ability to control material and cell positioning, appears as a promising approach to replicate the complexity of zonal variability in terms of cell densities and extracellular matrix (ECM) properties [31, 32]. Moreover, this technique offers other advantages, such as the possibility to reproduce subject-specific geometry and topography starting from medical images to create cell-laden constructs fitting to the defect of the specific patient [33].

In this review, we will describe how microfluidics and bioprinting can provide different insights in the field of mesenchymal stem cell-based cartilage repair and contribute to the development of novel therapeutic strategies. Specifically, since microfluidic and bioprinting technologies share the use of hydrogel-based materials, in the first section, we will focus on the optimization of these materials to mimic the composition and the mechanical properties of the articular cartilage. We will then describe the use of microfluidic devices for the identification of biochemical and biophysical factors driving stem cell chondrogenesis that could be implemented during the *in vitro* maturation of bioprinted constructs. In addition, we will describe studies whereby microfluidic and 3D bioprinting technologies have been applied to generate implantable constructs with a complex geometry. Finally, we will describe some new bioprinting applications that pave the way to the clinical use of stem cell-based therapies, such as scaffold-free bioprinting to generate clinically relevant constructs using 3D cell spheroids as building blocks and the development of a 3D biofabrication handheld device for the in situ repair of cartilage defects.

## 2. Microfluidics and Bioprinting to Trigger the Translation of Cell-Based Therapy

### 2.1. Advancements in Hydrogel-Based Materials

In several clinical applications, stem cells are directly injected into the target tissue without any biomaterial carrier. This process leads to limited stem cell engraftment at the treatment site, mainly due to leakage of cell suspension during injection [34]. Since the regeneration potential of stem cells is strongly correlated with the number of cells retained at the lesion site, improving stem cell engraftment is of utmost importance [34]. The use of hydrogel carriers has been introduced to overcome this limitation, by promoting cell retention at the desired site and providing the implanted cells with a microenvironment supporting cell viability and functions [34, 35]. In the context of cartilage regeneration, hydrogels have been widely applied, because of their numerous advantages. These highly hydrated polymeric networks that can be either natural or synthetic can be used for cell embedding as well as for incorporating growth factors and ECM components. Furthermore, hydrogels can be easily tailored in different geometries and, if properly designed, can provide the cells with an environment similar to that of native cartilage [36, 37].

Because of their intrinsic features, hydrogels have been applied as a 3D matrix for cell culture in microfluidic devices [38] as well as bioinks for 3D bioprinting. In particular, their increasing use in bioprinting has triggered the efforts in the optimization of hydrogel-based materials, in terms of composition, growth factor enrichment, and mechanical properties. Indeed, despite the promising advantages of bioprinting, one of the major challenges is the absence of a material that can be considered as the ideal bioink that satisfies all the specific requirements, as described in a number of recent reviews [39–42]. Regarding extrusion-based bioprinting process, the bioink should present shear thinning behavior to allow extrusion through the printer nozzle. At the same time, the bioink should be characterized by quick shear recovery to maintain the printed shape, showing adequate mechanical properties to guarantee a proper environment for embedded cells and long-term shape fidelity, manipulation, and ease of handling. Finally, the bioink should be biocompatible allowing long-term culture of bioprinted cells. The bottleneck of this technology is represented by the complexity to combine rheological/mechanical properties and biological properties, which are often mutually exclusive. To overcome this issue, there are two different approaches: either to improve the biocompatibility of materials characterized by adequate printing properties or to improve the printability of biocompatible materials [43]. For instance, Armstrong et al. [44] have formulated a new Pluronic-alginate multicomponent bioink with BMSCs to generate bone and cartilage structures. In this study, Pluronic was used as a sacrificial template in order to provide structural stability during printing, before chemical crosslinking of alginate took place, as well as to generate micropores and/or anisotropic microchannels in the construct to increase nutrient diffusion after its removal. Similarly, Kesti et al. [45] have improved the printability of a photocrosslinkable methacrylated hyaluronan (HAMA) by adding poly(N-isopropylacrylamide)-grafted hyaluronan (HA-pNIPPAM), a thermoresponsive polymer with good cytocompatibility used as a sacrificial template. In this way, the authors created a bioink in liquid state at room temperature that reticulates at body temperature. After thermal gelation, the biopolymer was stabilized through free radical polymerization of HAMA to achieve a long-term mechanical stability. Finally, the HA-pNIPPAM was eluted through medium washing at 4°C resulting in a glycosaminoglycan-based scaffold. This procedure allowed printing scaffolds with a diameter of 10 mm and a height of 2.8 mm, characterized by high biocompatibility as shown by articular chondrocyte viability.

The problem of the printability of soft biocompatible materials was also addressed by Müller et al. [46] developing a novel alginate sulfate-nanocellulose bioink for cartilage applications. Notwithstanding the innate biocompatibility of hydrogels derived from natural biopolymers such as hyaluronic acid, chitosan, or alginate, the rheological behavior of their solutions is often not suitable for 3D bioprinting. To overcome this problem, Müller and colleagues increased alginate viscosity through the addition of nanocellulose, changing also the behavior from Newtonian-like to shear thinning. The results of this study showed that articular chondrocytes embedded in alginate sulfate-nanocellulose were viable and synthesized type II collagen, proving the suitability of the newly developed biomaterial for cell-based cartilage repair. Composite bioinks combining nanofibrillated cellulose (NFC) with alginate (NFC/A) and hyaluronic acid (NFC/HA) were developed by Nguyen et al. [47]. In particular, in the case of cartilage, the NFC mimics the bulk collagen matrix, alginate simulates proteoglycans, and hyaluronic acid substitutes for the hyaluronic acid found in the native cartilage matrix. Noticeably, both alginate and nanofibrillated cellulose are xeno-free and FDA-compliant materials and hence can be easily translated into clinical use. The authors showed that both composite bioinks are printable; however, low proliferation and phenotypic changes of printed human-derived-induced pluripotent stem cells (iPSCs) were observed in the case of NFC/HA. On the other hand, iPSCs printed in NFC/A produced a relevant amount of hyaline-like cartilaginous tissue-rich and hyaline-like cartilaginous tissue-expressed chondrogenic markers, such as aggrecan. Differently from the aforementioned study [47] where the different constituents of the bioink mix resembled the different components of the articular cartilage, the approach developed by Levato et al. [48] to recapitulate the cartilage composition and architecture is based on the intrinsic ability of primary cells to produce specific ECM. In fact, these authors developed a zonal-like model using two different cell sources, chondrocyte progenitor cells (CPCs) and BMSCs encapsulated in gelatin methacrylamide (GelMa) hydrogels. By combining CPC- and MSC-laden bioinks, a bioprinted model of the articular cartilage was generated, consisting of defined superficial and deep regions, each with distinct cellular and ECM composition. Noticeably, the authors showed that their bioprinting method, which uses Pluronic F-127 as sacrificial ink to support GelMa during the biofabrication process, allows fabricating clinically relevant anatomical structures. However, to match the mechanical properties of the articular cartilage, the bioprinted material should undergo an extensive *in vitro* maturation before implantation or reinforcement of a supporting material. Indeed, hydrogels show low compressive stiffness resulting to becoming unsuitable for application in the fabrication of load-bearing tissues. For this reason, several strategies have been exploited to reinforce hydrogels using stiffer materials [27]. For instance, Daly et al. [49] engineered mechanically reinforced hydrogels by codepositing soft bioinks, such as agarose, alginate, and GelMa, with polycaprolactone (PCL) filaments. In this way, the authors were able to obtain BMSC-laden constructs with bulk compressive modulus similar to the native articular cartilage. Noticeably, this approach allowed at the same time enhancing the printability of soft hydrogels and matching the mechanical properties needed to withstand high mechanical loading within a joint environment, a result that is hardly achievable when using standard hydrogels [23]. Another interesting work that focuses on the reinforcement of bioinks was performed by Kang et al. [50] who developed an integrated tissue-organ printer (ITOP). This device includes a multiple cartridge system that allows the deposition of cell-laden composite hydrogels in combination with PCL polymer and an external sacrificial Pluronic F-127 hydrogel, which reinforces the material properties and supports the structure, respectively, during printing. Through a 3-axial motorized stage system and an air pressure-based controller, the ITOP is able to precisely regulate the dispensing volume of each material enabling the production of constructs with structural integrity and complex geometry. The promising results obtained using this system to generate a human-scale ear-shaped bioprinted construct indicate that this approach would be also suitable to print clinically relevant constructs recapitulating the structures and features of the native articular cartilage.

**Figure 1:**
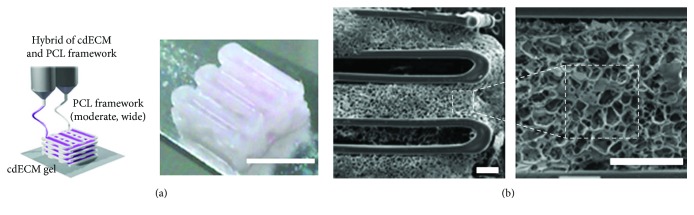
Bioprinting of 3D constructs using a bioink based on a tissue-specific decellularized matrix. (a) Bioprinting process for the obtainment of hybrid structures made of decellularized cartilage ECM (cdECM) and PCL for cartilage repair. Scale bar = 5 mm. (b) SEM images of the bioprinted construct. Scale bars = 400 *μ*m (adapted from [52]).

Despite the aforementioned approaches, the formulation of the optimal bioink for cartilage tissue regeneration is still to be achieved. Furthermore, the intrinsic complexity of the native ECM is often neglected in 3D bioprinting experiments. It is well known that the ECM microenvironment plays a key role in directing the differentiation of stem cell through receptor ligand interactions and mechanotransduction [51]. Hence, to successfully generate an instructive cell niche, the tissue-specific cell-ECM interactions have to be recapitulated. To this purpose, Pati and colleagues [52] have recently proposed the use of a decellularized extracellular matrix (dECM) as bioink to maintain the complexity of the native tissue (Figure 1). In particular, the articular cartilage was decellularized, solubilized, combined with inferior turbinate tissue-derived mesenchymal stromal cells (TMSCs), and printed with a layer-by-layer technique using a PCL polymeric framework to support the structure during printing and gelation. The obtained results showed that dECM scaffolds provided a biocompatible microenvironment for cell proliferation and outperformed the control materials in directing tissue-specific lineage commitment, as revealed by the increased expression of chondrogenic markers. Remarkably, this study demonstrated that bioprinting with dECM bioink is an attractive option that paves new ways for both *in vitro* and *in vivo* tissue reconstruction. Additionally, the developed material could be also particularly beneficial in the field of *in vitro* cartilage models that currently use standard and nonspecific hydrogels, such as type I collagen and fibrin, as a 3D matrix for cell culture. For instance, the dECM hydrogels, gelling at 37°C, could be easily used in microfluidic models to better mimic the chondral environment and to provide the cells with an instructive cell niche, representing a significant step forward in the development of biomimetic chondral models. This study perfectly shows how the efforts in a research field strongly related to the *in vivo* application, such as 3D bioprinting, can lead to important advancements in other apparently unrelated fields, such as *in vitro* biomimetic models, representing a perfect example of the convergence between different scientific areas.

### 2.2. Evaluation of Biochemical and Biophysical Factors

**Figure 2:**
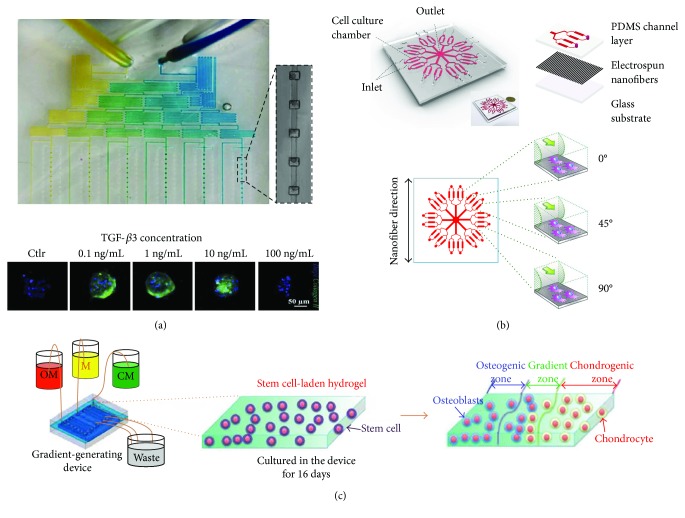
Microfluidic models for the screening of biochemical and biophysical factors. (a) Photograph of a microfluidic device including a serial dilution generator to develop a gradient of growth factors and a 3D culture area for the generation and perfusion of 3D cell spheroids. This device was exploited to generate a gradient of TGF-*β*3 and to identify the concentration able to induce type II collagen expression in micropellets (adapted from [16]). (b) Schematic illustration of a microfluidic device with integrated electrospun nanofibers to study the influence of 3 different flow directions with respect to fiber orientation on stem cell chondrogenesis (adapted from [58]). (c) Schematic representation of a microfluidic device for the development of the osteochondral interface. A system formed by two serpentines and a central channel, respectively, filled with osteogenic medium (OM), chondrogenic medium (CM), and cell culture medium (M), allows the generation of osteogenic and chondrogenic growth factor gradient to obtain spatially controlled differentiation of MSCs (adapted from [59]).

Stem cell differentiation protocols exploit developmental signals to instruct the cells and drive them towards a specific lineage. The generation of models for the screening of multiple growth factors is a crucial step to define differentiating signals able to recapitulate *in vitro* the developmental processes leading to chondrogenesis *in vivo*. Indeed, the identification of biochemical and biophysical parameters able to trigger stem cell chondrogenesis can significantly impact the design of proper culture conditions for *in vitro*-generated constructs in terms of medium composition as well as the development of dynamic culture systems for the application of biophysical stimuli. In this scenario, the results obtained using *in vitro* models could be implemented during the *in vitro* maturation of biofabricated constructs before their implantation, in order to achieve the chondrogenic priming of the engineered tissue. The definition of the most suitable combination of differentiating signals often requires the screening of several growth factor combinations, as well as concentration ranges and timing, which easily results in a complex experimental setup based on many levels of interactions among multiple parameters. In this context, microfluidic technology offers several advantages related to the minimal number of cells required to test a high number of experimental conditions and the use of very low amounts of costly growth factors. Even more importantly, microfluidic models feature an unprecedented spatial and temporal control over the cell microenvironment. Indeed, the controlled perfusion of the culture medium within microchannels allows maintaining more uniform culture conditions than standard static approaches, providing the stable supply of nutrients and growth factors as well as the removal of waste products [13, 53]. Remarkably, controlled fluid flow can also be exploited to automatically obtain gradients of growth factors in the same microfluidic platform using serial dilution generators (SDGs). This strategy was recently applied by Occhetta et al. [16] who developed a microfluidic platform implementing two different SDGs to generate either a wide range of concentrations of soluble factors (logarithmic scale) or a narrower concentration window (linear scale) (Figure 2(a)). This device was specifically designed to induce the condensation of BMSCs within fluidically connected microchambers, enabling the formation of 3D micropellets with uniform size and shape. Using this platform, the authors were able at the same time to uniformly generate 3D cell micropellets into defined spatial configurations and culture them under a continuous laminar flow with defined concentrations of transforming growth factor- (TGF-) *β*3 spanning over four orders of magnitude. This screening led to identify the lowest TGF-*β*3 concentration (0.1 ng/mL) as capable of inducing chondrogenesis and maintaining the proliferative ability of BMSCs, while the highest TGF-*β*3 concentration (100 ng/mL) induced the disaggregation of the micropellets. Noticeably, the results of this study demonstrated that the developed model allows replicating the 3D expansion step occurring during the early stages of limb development, overcoming one of the main limitations of other 3D models, such as macropellets, which experience a reduction in cell number over time due to the formation of a necrotic core [54]. The use of SDGs has been also implemented in microfluidic platforms designed and exploited to select the most suitable concentration and/or combination of growth factors to favor articular chondrocyte proliferation, either in monolayer culture [55] or hydrogel-based culture [56]. The low number of cells that can be obtained from patients' biopsies is one of the main limiting factors in ACI procedures, and hence, the optimization of the protocol for chondrocyte expansion represents a crucial step to improve the outcome of this clinical approach. Another strategy to overcome this limitation is represented by combining MSCs and articular chondrocytes as proposed by Higuera et al. [57] who developed an implantable screening device that allows the analysis of multiple coculture conditions both *in vitro* and *in vivo*. The device consists in a 3D-printed platform formed by arrays of micro- to millimeter-scale wells that can be subcutaneously implanted in nude mice to evaluate the influence of the cell source on the accumulation of ECM. Because of this system, the authors identified the optimal ratio of MSCs and articular chondrocytes to achieve cartilaginous ECM deposition, demonstrating the existence of optimal conditions for the crosstalk between these two cell types.

Besides biochemical factors, the role of biophysical factors as determinants in stem cell differentiation is also gaining more and more attention. The development of a biomimetic microenvironment allows a more precise study of cell behavior in physiological-like conditions. In this scenario, microfluidics has major advantages compared to conventional 2D culture due to the possibility of replicating some aspects of the *in vivo* 3D environment in terms of both biochemical and physical stimuli. In this context, Rivera and Baskaran [17] have developed a microfluidic device to investigate simultaneously the influence of shear stress and biomolecular gradients on BMSC alignment and chondrogenesis. The 3-week exposure to TGF-*β*1 gradients via fluid flow enhanced the formation of chondrogenic aggregates with an increased cell elongation in flow direction when compared to a constant TGF-*β*1 concentration. The cellular alignment was more evident in confluent regions with respect to nonconfluent regions demonstrating that both shear stress and cell-cell contact influenced BMSC behavior. Hence, exposing BMSCs to gradients of chondrogenic factors and shear stress appears as a promising strategy to induce chondrogenesis that can be implemented to generate engineered tissues with superior features compared to standard culture. An alternative microfluidic approach was exploited to investigate the effect of sequential mechanical and biochemical stimuli on stem cell differentiation [18]. To this purpose, a shear stimulation system controlled by a syringe pump was used to apply a physiological shear stress of 1.5 Pa to a suspension of BMSCs flowing through the tube. Shear-stimulated BMSCs were then exposed to biochemical factors to determine if preconditioning BMSCs with a mechanical stimulus could enhance their response to chondrogenic factors. Remarkably, this study highlighted that BMSCs retain the memory of a single shear stimulation experience and that 3 weeks later, the commitment towards the chondrogenic lineage was still superior in shear-stimulated compared to nonstimulated cells, demonstrating the potential of this approach to influence stem cell fate and responsiveness to differentiating factors in a short timeframe.

Recent advances in microfluidic technology have also allowed the incorporation of nanostructures in 3D microfluidic models to create a more biomimetic microenvironment and understand the mechanotransduction dynamics modulating stem cell fate. In particular, Zhong et al. [58] integrated aligned nanofibers obtained by electrospinning into a microfluidic platform to investigate the simultaneous role of topographical cues and mechanical cues provided by fluid flow (Figure 2(b)). The authors designed a microfluidic device containing independent microchambers with multiple orientations with respect to electrospun nanofibers to allow fluid flow to form different angles with the nanofibrous substrate. The results showed that BMSCs preferentially elongated along the nanofiber direction and that chondrogenesis was improved by the presence of perpendicular flow. In this condition, the expression of type II collagen was higher, whereas a significant increase of type I collagen was observed in cells under parallel flow, demonstrating how specific mechanotransduction signaling pathways regulate BMSC differentiation by translating mechanical stimuli into biochemical signals.

From the perspective of stem cell-based restoration of the articular cartilage, the simultaneous triggering of efficient chondrogenesis and osteogenesis of stem cells in the spatially defined region of a 3D scaffold appears to be a promising strategy to develop *in vitro* models for the study of bone-cartilage crosstalk and, hence, to achieve new strategies to improve the integration of the chondral graft. In this context, the microfluidic technology provides powerful tools to engineer interfacial tissues by exploiting the intrinsic multidifferentiation ability of stem cells. These constructs can be either used as a disease model to investigate the role of pathogenic signals that may affect both cartilage and the subchondral bone or to generate implantable constructs, representing a *trait d'union* between *in vitro* and *in vivo* applications. Recently, Shi and colleagues [59] have developed a microfluidic system able to generate gradients of chondrogenic and osteogenic growth factors to steer the spatially controlled chondrogenesis and osteogenesis of ADMSCs embedded in the same hydrogel (Figure 2(c)). This gradient-generating system includes a bottom layer consisting of a PDMS pool filled with a stem cell-laden agarose hydrogel covered with a microporous membrane and with a top layer containing two lateral serpentine channels and a central linear channel. To produce a biomimetic transitional phase between osteogenic and chondrogenic zones, a culture medium without any differentiation factors was introduced into the central channel while chondrogenic and osteogenic media were introduced into the two lateral serpentines. After 25 days of culture, the spatially controlled differentiation of stem cells into chondrocytes and osteoblasts was achieved and a region mimicking the bone-cartilage interface was observed in the central region of the hydrogel. The implementation of this type of gradient-generating system in a more complex and clinically relevant setup may pave the way to the *in vitro* engineering of interfacial tissues starting from a single cell source seeded in a single biomaterial. This platform could also be used to study the pathogenesis of diseases involving both the articular cartilage and the subchondral bone, such as OA. Indeed, the generated construct may represent a reliable *in vitro* model of the osteochondral interface and the two lateral serpentines could be used to generate a gradient of pathogenic signals (e.g., proinflammatory cytokines) starting from either the chondral or the bony side. A similar approach has been used by Lin and colleagues [60] who developed a microphysiological model of the osteochondral unit integrating a microfluidic system into a multichamber bioreactor. This system was exploited to achieve spatially defined chondrogenic or osteogenic differentiation of BMSCs loaded in a methacrylated gelatin-based scaffold and to induce an OA-like response through the targeted treatment of either the chondral or the bony compartment with the proinflammatory cytokine interleukin- (IL-) 1*β*. Using this approach, the authors demonstrated that the exposure of the bony layer to IL-1*β* resulted in a stronger catabolic response in the chondral layer than the direct application of IL-1*β* to the chondral component, indicating the active communication between the two tissues. 3D bioprinting techniques have been also exploited to generate anisotropic microscale multiphase 3D tissue models with potential impact in *in vitro* drug testing, discovery, and development as reported by Gurkan et al. [61]. In this study, the generation of an interfacial tissue was achieved by printing BMSCs in nanoliter hydrogel droplets encapsulating either bone morphogenetic protein- (BMP-) 2 or TGF-*β*1 to drive their differentiation towards different lineages. The authors showed that phenotypic pathway and network analysis can be performed using the genomic expression data obtained from the model, demonstrating the potential of bioprinted anisotropic tissues as functional *in vitro* 3D tissue models.

### 2.3. Improvement of Construct Architecture

In stem cell-based cartilage applications, the use of hydrogel biomaterials has been introduced to improve cell retention at the injury site and to provide the implanted cells with a favorable microenvironment. However, the use of bulk hydrogels has some disadvantages including a high risk of ectopic chondrogenesis and an inefficient supply of oxygen and nutrients due to the limited diffusion within hydrogels, which is often restricted to 200 *μ*m and results in a necrotic core [62].

**Figure 3:**
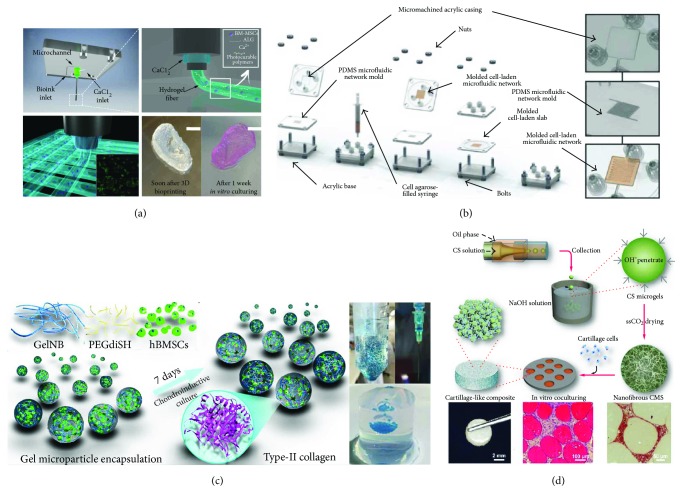
Application of microfluidic and bioprinting technologies for the development of 3D cartilaginous constructs. (a) Schematic representation of the custom-made dispensing coaxial system: calcium chloride flows in the external nozzle while the bioink is supplied through the inner one. Hydrogel fibers form immediately at the tip of the inner nozzle when the two solutions come into contact. Real-size neonatal ear can be obtained with a high printing resolution (≈100 *μ*m). Scale bar = 10 mm (3D bioprinting method reported in [71]). (b) Fabrication process of a cell-laden agarose construct with an incorporated microfluidic serpentine to enhance oxygen and nutrient transport (adapted from [66]). (c) Schematic diagram of chitosan microsphere production through a coaxial glass microcapillary device. Each nanofibrous microsphere is seeded with articular chondrocytes, and the deposition of newly generated ECM tightly bridges the microspheres into a clinically relevant 3D construct (adapted from [21]). (d) Schematic representation of hydrogel microspheres with encapsulated BMSCs produced by a simple syringe-based system. The obtained microspheres can be injected into the lesion site, as demonstrated by the injection into an agarose gel model, which mimics an articular cartilage defect (adapted from [20]).

With advances in engineering technologies, such as soft lithography and 3D bioprinting, microfluidic channels and complex geometries have been engineered into hydrogels to improve perfusion for the delivery of oxygen and nutrients and removal of metabolic waste products for the embedded cells [19, 63–67]. To obtain printable microfluidic channels, Zhang and coworkers [19] have combined 3D bioprinting and microfluidics for the generation of cell-embedding hollow filaments. In this study, coaxial nozzles were fabricated using three fluid-dispensing tips and assembling a feed tube, an outer tube, and an inner tube. The feed tube was used to deliver the alginate solution into the cavity formed between the outer and inner tubes, while the CaCl_2_ crosslinking solution was fed through the inner tube to create the hollow filament. By modulating the flow rate of alginate and CaCl_2_ solutions, the authors were able to tune the ratio between the core diameter and the fiber diameter demonstrating the great flexibility of this technique. Chondrocyte progenitor cells (CPCs) encapsulated within the hollow alginate fibers showed a high cell viability, proving the cytocompatibility of this process. Noticeably, the expression of chondrogenic markers was enhanced in encapsulated CPCs compared to monolayer culture, indicating that alginate hollow filaments provide an ideal environment for CPCs to differentiate and carry out their cartilage-producing function. This strategy allows fabricating 3D constructs loaded with progenitor cells, yielding the viability and functionality of the cells seeded in the central region, which usually have limited access to nutrients and oxygen. Furthermore, as envisioned by the authors, this approach can be implemented by printing CPC spheroids between filaments and pumping a culture medium through the hollow channels to promote the formation of a cartilage-specific matrix in tissue constructs with a clinically relevant size. As aforementioned, the insufficient supply of nutrients and oxygen and the inefficient waste removal are major disadvantages when engineering in vitro 3D thick tissues. Embedding microfluidic networks within 3D hydrogel scaffolds represents a promising approach to improve perfusion through thick tissues. Choi and coworkers [65] have presented a strategy to control the distributions of soluble chemicals within the scaffold with convective mass transfer via microfluidic networks embedded within the cell-seeded biomaterial. The authors exploited a lithographic technique to generate functional microfluidic serpentines in a calcium alginate hydrogel seeded with articular chondrocytes and characterized convective and diffusive solute transfer, demonstrating that microfluidic channels enable efficient exchange of solutes with the bulk of the scaffold and quantitative control of the soluble signals experienced by the cells. This approach was also suitable to generate two independent microfluidic networks in the same scaffold, which could be particularly relevant in view of the administration of different growth factors to induce the spatially controlled differentiation of stem cells seeded within the same hydrogel to engineer interfacial tissues. A similar strategy was adopted by Goldman and Barabino [66] to design agarose constructs embedding a microfluidic serpentine in order to enhance the viability of encapsulated articular chondrocytes and the production of type II collagen and glycosaminoglycans (Figure 3(a)). To this purpose, a PDMS mold was used to generate a cell-laden agarose layer integrating a microfluidic serpentine (425 × 425 *μ*m square cross-section) that was then sealed against a planar slab of a cell-laden agarose solution to complete the construct. This study showed that the incorporation of a microfluidic network in cell-laden agarose gels allows improving proliferation and ECM biosynthesis in tissue-engineered constructs of relevant thickness (2.5 mm and 5 mm thick) compared to bulk hydrogels.

Considering the exploitation of microfluidics as a biofabrication technology, interesting studies have been recently published whereby microfluidics was used to produce 3D scaffolds with uniform pore sizes [68, 69]. Specifically, Chung et al. [68] used a microfluidic device including two concentric tapered channels to generate bubbles enclosed within liquid alginate droplets by pumping nitrogen gas and aqueous alginate solution through the inner and the outer channels, respectively. These bubbles spontaneously self-assembled into a liquid foam that was then exposed to a CaCl_2_ solution to induce alginate crosslinking and generate a solid foam. This approach generated scaffolds with highly ordered and interconnected pores with controlled size. In a following study [69], the same group showed that this honeycomb porous scaffold, which is characterized by a more ordered structure than traditional alginate sponges, well supported chondrocyte growth and phenotype maintenance demonstrating that this highly organized scaffold prepared with an economical microfluidic device holds potential for future applications in the field of cartilage tissue engineering.

The recent combination of 3D bioprinting with advanced microfluidic printheads has recently found application in many areas, leading to unprecedented advances in the biofabrication of complex tissue constructs with high spatial resolution [70]. In the context of cartilage repair, a system based on two coaxial needles has been used to fabricate 3D scaffolds via bioprinting composed of ECM biomimetic hydrogels loaded with BMSCs (Figure 3(b)) [71]. In details, the authors have developed a bioprinting system formed by an external nozzle and an inner nozzle, dispensing CaCl_2_ and different alginate-based hydrogel solutions, respectively. In this way, as the two solutions came into contact, hydrogel fibers formed immediately at the tip of the inner nozzle through a gelation process that allowed producing 3D hydrogels with high resolution. After 3D bioprinting, the constructs underwent a secondary UV crosslinking to guarantee an efficient bonding among fibers belonging to adjacent layers determining the overall mechanical properties of the scaffolds. In particular, structures with a 5 mm height were printed depositing 50 layers with 100 *μ*m thickness. 3D bioprinting experiments performed with BMSCs showed that ionic crosslinking of alginate and UV crosslinking were not detrimental to cell survival, proving the biocompatibility of this approach.

Microfluidic technology has been also exploited to achieve cell microencapsulation generating cell-laden microgels in a high-throughput manner, as reported in several recent reviews [72–74]. Remarkably, the use of these microgels as building blocks that can be combined to obtain relevant constructs offers a major advantage with respect to bulk hydrogels, since the large surface-to-volume ratio promotes a more efficient mass transport and enhanced cell-matrix interactions. In the context of cartilage repair, Li and colleagues have developed a simple and cheap microfluidic device to encapsulate BMSCs in hydrogel-based microspheres that can be crosslinked using visible light [20] (Figure 3(c)). Specifically, the device was composed of an ordinary pipette tip and two tubes connected to two syringes: one containing a precursor hydrogel solution (aqueous phase) and BMSCs and the other one loaded with oil (oily phase). First, the pipette chamber was filled with oil, and then, the dispersed hydrogel phase was pumped into the tube at a constant rate to generate the microspheres through the silicone tube. In this way, the authors were able to generate microspheres with different diameters (ranging from 300 to 600 *μ*m) by adjusting the flow rate ratio between the aqueous and the oily phase. The authors showed that BMSCs encapsulated into the microspheres achieved a superior chondrogenesis compared to the bulk hydrogel and that microspheres could be injected into a cavity simulating a focal cartilage lesion with a 20-gauge hypodermic needle, thus proving the feasibility of intra-articular microsphere injection and the clinical relevance of this approach.

Microfluidic technology has also been exploited as a manufacturing technique for the preparation of microparticles to generate clinically relevant 3D constructs, as reported by Zhou and colleagues [21] (Figure 3(d)). Specifically, a coaxial glass microcapillary device was assembled by round and square glass capillaries, and chitosan and cyclohexane solutions were flown to generate an inner aqueous and an outer oil phase, respectively. The generated emulsion was collected in an alkaline solution to induce the gelation of chitosan microspheres (*ϕ* 165–425 *μ*m), which were then seeded with articular chondrocytes and cultured for 7 days. The authors showed that this period was sufficient for articular chondrocytes to tightly bridge chitosan microspheres through ECM into bigger aggregates that were then transferred into molds (*ϕ* 5 mm, *h* 2 mm) and cultured in static conditions for 14 additional days. At the end of culture, histological analysis showed that the spaces among the microspheres were filled with cartilage-specific ECM rich in glycosaminoglycans. Furthermore, the generated constructs were able to withstand several compression cycles and displayed a certain degree of elasticity, indicating that the microspheres were tightly bonded together by the chondrocytes and the secreted ECM and demonstrating the validity of this bottom-up approach for cartilage tissue engineering applications.

### 2.4. New Approaches towards Clinical Practice

**Figure 4:**
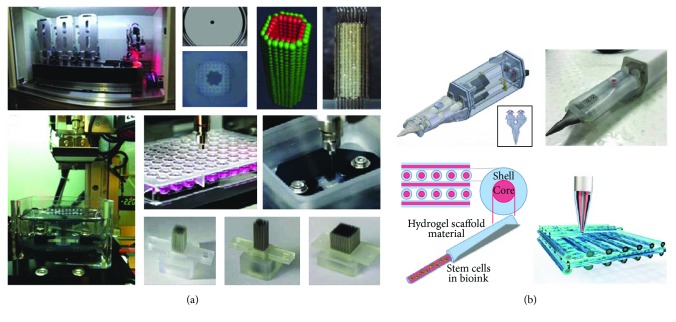
3D bioprinting approaches towards the clinical translation of cell-based therapies. (a) Kenzan Method, based on an automated system for cell spheroid bioprinting, allows the deposition of 3D spheroids on a microneedle array as a support for the production of scaffold-free 3D constructs [75]. (b) Schematic illustration of Biopen, a handheld device constituted of two bioink chambers, a collinear nozzle, and a UV crosslinking source for the in situ deposition of cell-laden methacrylated hydrogels (adapted from [79]).

A particular 3D bioprinting approach to obtain highly organized constructs for tissue regeneration has been invented by Professor Nakayama, using cell spheroids as building blocks (Figure 4(a)) [75]. This innovative method belongs to the biomaterial scaffold-free approach, for which exogenous materials are not required. Differently from previous studies whereby spheroids were manually assembled into 3D clinically relevant constructs using cylindrical molds [76, 77], in the “Kenzan Method,” a 3D bioprinter is used to robotically place cell spheroids in microneedles, which are used as a temporary support during the fusion of spheroids. Each array is composed by 160 *μ*m thick microneedles, which are 500 *μ*m distant from each other, and therefore, spheroids should have a diameter of a hundred micrometers to get in contact and form ECM in order to achieve a compact construct. After the spheroid fusion, the constructs are removed from the needle support and cultivated for the postprinting maturation phase in which the holes formed by the needle are resorbed due to their cell “healing” capacity. Remarkably, although this technique differs from the standard approach of 3D bioprinting for the presence of preformed cell spheroids and for the absence of hydrogel-based materials, it represents a valid method to produce constructs with a clinically relevant size, avoiding potentially detrimental processes, which can occur during standard 3D bioprinting procedures.

Another interesting approach that shares some fundamental principles with 3D bioprinting, such as the use of living cells and biomaterials as building blocks, is represented by a 3D bioprinting pen, called “Biopen”, which was developed by O'Connell et al. [78]. This new approach, which represents one of the most relevant applications in view of the clinical translation of bioprinting, was designed to overcome the issues related to the traditional procedure for tailoring implants to the anatomy of the defect. This process involves the use of medical imaging data to create implant design before the chondral repair procedure. However, such a method does not take into account the initial steps of surgery in which the surgeon removes the excess of fibrous tissue around the defect, thus varying its size and shape. Differently from the standard bioprinting approach, Biopen does not use a CT/MRI image for the development of a digital model of the defect to direct the deposition of cells and biomaterials, but the bioprinting process is manually operated by the user in a direct writing fashion during the surgical procedure. This feature that represents the major difference between the Biopen approach and standard 3D bioprinting is also the main advantage of this device allowing the fabrication of constructs that perfectly fit the shape and size of the chondral defect. This handheld fabrication tool is composed of three main components: an inner 3D-printed core that contains two collinear ink chambers, a custom titanium extruder nozzle, and a UV source. The extrusion process is controlled by the user through a foot pedal-based pneumatic system that allows depositing each ink individually and/or simultaneously. In a preliminary phase, Biopen has been tested in 2D deposition processes to verify the printing stability and the ability to create compositional gradients controlling the relative extrusion rates of the two chambers. The obtained results demonstrated the printing capacities and consistency of fabricated objects using a GelMa/HAMA hydrogel. In the latter phase, biological experiments have been performed to evaluate the effects of the printing process on ADMSCs. In a subsequent work, the same research group performed a pilot study to evaluate the surgical applicability of Biopen to repair critical full thickness chondral defects (*ϕ* 8 mm) in an ovine model [79]. Differently from the above-described device, the new version of Biopen is characterized by a coaxial extrusion system that allows depositing a biphasic hydrogel constituted by an inner “core” of GelMa-HAMA bioink laden with ADMSCs from infrapatellar fat pad and an outer “shell” of GelMa-HAMA bioink mixed with the photoinitiator (Figure 4(b)). The presence of this outer shell warrants photocuring of the construct during printing. The obtained results showed that Biopen was able to deliver 3D-printed scaffolds perfectly fitting the shape and depth of the defect, without causing any sign of inflammation or infection. Moreover, constructs printed through Biopen showed a higher amount of newly generated cartilage if compared with both negative untreated controls and defects treated by microfracture technique, evidencing chondrocyte columnar alignment and maintenance of subchondral bone integrity after 8 weeks from implantation. Promising results were obtained also evaluating the mechanical properties of Biopen-extruded scaffolds, which yielded values of instantaneous Young's modulus, equilibrium modulus, and maximum stress similar to those of the native articular cartilage. The promising outcomes reported in these studies [78, 79] and the recent results regarding the optimization of the bioprinting conditions to achieve high cell viability and relevant structural stiffness [80] may pave the way to the use of Biopen to build up mm- to cm-scale 3D structures. More importantly, because of its ability to directly control the deposition of biomaterials during the surgical process, this device can represent an exciting advance in the translation of bioprinting into clinical practice, not only for cartilage regeneration but also in other applications where tissue regeneration is critical.

## 3. Outlook

Although stem cell-based therapies have emerged as a novel treatment in cartilage-based repair, their success is often limited due to multiple factors, such as inefficient differentiation of stem cells towards the chondrogenic lineage and/or poor stem cell engraftment and survival after transplantation. In addition, an important aspect that is often neglected is that stem cells are usually delivered to an inflamed environment and, hence, have to face a plethora of catabolic signals that may negatively affect the outcome of cell-based approaches.

In this scenario, microfluidics can provide important advances in the selection of biochemical and biophysical factors able to direct the fate of stem cells that can be implemented in new protocols for stem cell differentiation and in the design of dynamic culture systems. It is also possible to envision an exploitation of microfluidic models personalized with patient-derived stem cells for the screening of the most suitable differentiation protocol for each patient-specific stem cell population. This would allow the optimization of personalized differentiation protocols. Additionally, since the use of growth factors during *in vitro* culture may pose obstacles in the clinical translation of stem cell-based therapy, the possibility to direct stem cell fate uniquely using biophysical factors, such as shear stress, appears to be fascinating. Microfluidic models hence represent an invaluable tool to define the biophysical stimuli that should be used to direct stem cells towards the chondrogenic lineage without using growth factors. Furthermore, microfluidic devices can be used to develop organotypic models of the whole articular joint capable to recapitulate either a physiological or a pathological environment [81], perfectly matching the concept of organs-on-chips for the study of tissue development, organ physiology, and disease etiology [82, 83]. The models that recapitulate the osteochondral unit on a single chip are a striking evidence of the potential of microfluidics in this context. Indeed, the comprehension of the inflammatory events involving the articular cartilage and subchondral bone may help in defining complementary anti-inflammatory treatments to promote the survival and the engraftment of implanted stem cells. Recently, a new strategy to mold and culture composite 3D cellular constructs featuring different cell types and/or biomaterials, with high spatial control in microfluidic channels, has been developed [84]. This technique allows obtaining a continuous gel-gel interface, with no need for pillars to delimit the hydrogels, and paves the way to the development of microfluidic models including chondral and osseous compartments with highly specific features in terms of cell and ECM composition. Using this approach, it would be possible to combine a miniaturized model of the articular cartilage with a miniaturized model of the subchondral bone, including a calcified ECM, osteoblasts, osteoclasts, and endothelial cells [85]. Bioprinting could be also used to generate microfluidic models of the articular joint, since it enables the printing of multiple materials and different types of living cells in a programmable manner with high spatial resolution, and has proven to hold a great potential for fabricating organs-on-a-chip recapitulating the intrinsic complexity of the native tissue/organ [86, 87].

On the other hand, microfluidics-based 3D bioprinting approaches could be used to overcome the problem of poor cell engraftment at the lesion site, by transplanting stem cells embedded in an ECM-mimicking environment. 3D bioprinting is rapidly becoming a first-choice approach for several advanced applications in tissue engineering. Despite the appealing advantages offered by this technology, such as rapid production of cellularized constructs with high accuracy and repeatability independently of scaffold geometry, a major ongoing challenge that still needs to be addressed is related with bioink formulation. In fact, an ideal bioink should provide, on one side, a proper matrix for cell maturation, differentiation, and neomatrix synthesis while still keeping, on the other side, a high printing resolution. So far, this issue has been addressed only to a minor extent and a continuous research is carried out to find new solutions. In the case of cartilage regeneration, 3D bioprinting represents a suitable technology to recapitulate the tissue structure in terms of chondrocytes and ECM organization. In particular, the complex zonal organization might be reproduced in the future through the development of more accurate systems for multimaterial deposition. Furthermore, the formulation of bioinks should be refined to better promote stem cell differentiation towards the chondrogenic lineage with the synthesis of new polymers or with the formulation of blends or composite bioinks that would eventually result in enhanced quality of the neodeposited matrix. Another major issue that must be overcome in order to boost the translation of engineered constructs for cartilage regeneration into the clinic is related to their poor mechanical properties. In fact, native cartilage has a Young's modulus of around 700–800 kPa, which is between one and two orders of magnitude higher compared to the constructs obtained via 3D bioprinting. This is a key issue that would require many efforts to be overcome. A possible solution may be found by employing more sophisticated culture systems that may lead to more functional cartilage tissue by providing controlled mechanical and biochemical stimuli. However, then, we need to be sure that biofabricated mature tissue will properly integrate with surrounding natural cartilage. So far, 3D bioprinting has already demonstrated its capacity to build complex artificial structures. However, the future work must be focused on enhancing the functionality of such constructs to prompt applications in real clinical scenarios. Finally, in situ 3D bioprinting can enable the achievement of thick tissues directly into the lesion site in a one-step approach, by translating bioprinters in the surgery room. Despite challenges, this computer-aided technology holds a great potential since it would allow overcoming the need for preshaping or reshaping of the scaffold based on the defect geometry and achieving high precision in the deposition of cells and biomaterials. Because of these features, we envision that in situ 3D bioprinting will produce significant advances in the regeneration of the entire articular units or in the treatment of complex articulations, such as the carpometacarpal joint.
